# Genetic mapping and QTL analysis of Botrytis resistance in *Gerbera hybrida*

**DOI:** 10.1007/s11032-016-0617-1

**Published:** 2017-01-23

**Authors:** Yiqian Fu, Alex van Silfhout, Arwa Shahin, Ronny Egberts, Martin Beers, Ans van der Velde, Adrie van Houten, Jaap M. van Tuyl, Richard G. F. Visser, Paul Arens

**Affiliations:** 1 Plant Breeding, Wageningen University & Research, PO Box 386, 6700AJ Wageningen, The Netherlands; 2 Schreurs Holland B.V., Hoofdweg 81, 1424PD De Kwakel, The Netherlands; 3 Florist Holland B.V., Dwarsweg 15, 1424PL De Kwakel, The Netherlands

**Keywords:** Gerbera grey mould, SNP, Linkage group, QTL mapping

## Abstract

*Gerbera hybrida* is an economically important cut flower. In the production and transportation of gerbera with unavoidable periods of high relative humidity, grey mould occurs and results in losses in quality and quantity of flowers. Considering the limitations of chemical use in greenhouses and the impossibility to use these chemicals in auction or after sale, breeding for resistant gerbera cultivars is considered as the best practical approach. In this study, we developed two segregating F1 populations (called S and F). Four parental linkage maps were constructed using common and parental specific SNP markers developed from expressed sequence tag sequencing. Parental genetic maps, containing 30, 29, 27 and 28 linkage groups and a consensus map covering 24 of the 25 expected chromosomes, could be constructed. After evaluation of *Botrytis* disease severity using three different tests, *whole inflorescence*, *bottom* (of disc florets) and *ray floret*, quantitative trait locus (QTL) mapping was performed using the four individual parental maps. A total of 20 QTLs (including one identical QTL for whole inflorescence and bottom tests) were identified in the parental maps of the two populations. The number of QTLs found and the explained variance of most QTLs detected reflect the complex mechanism of *Botrytis* disease response.

**Electronic supplementary material:**

The online version of this article (doi:10.1007/s11032-016-0617-1) contains supplementary material, which is available to authorized users.

## Introduction

*Gerbera hybrida* belongs to the Compositae family and is known for its abundant flower colours, capitula sizes and shapes. As one of the most economical important ornamental plants, gerbera is mainly used as cut flower and ranked fifth in cut flower sales on Dutch flower auctions in 2014 (https://www.floraholland.com/media/3949227/Kengetallen-2014-Engels.pdf). The cultivated commercial gerberas are highly heterozygous, almost completely obligatory outcrossing diploid (2*n* = 50) plants and probably originated from crossings between two wild species, *Gerbera jamesonii* and *Gerbera viridifolia*, from Africa (Hansen 1999).

Gerbera as a cut flower is mainly grown in greenhouses for year-round production. However, during gerbera cultivation especially in winter and during the process of post-harvest transportation, the high relative humidity is ideal for grey mould infestation. Gerbera grey mould is caused by the necrotrophic fungus *Botrytis cinerea*, a notorious fungal pathogen with a wide range of plant host species (Elad et al. 2016). *B. cinerea* infection leads to direct damage on gerbera. Necrotic lesions (spotting) in early infection occur on flower buds and ray florets and these symptoms are strengthened when flowers are packed in boxes, in which a high relative humidity develops during cold storage and transport (Bastiaan-Net et al. 2007; Kerssies 1993a; Kerssies 1993b; Salinas and Verhoeff 1995). Control of gerbera grey mould in greenhouses frequently relies on spraying chemicals (Prins et al. 2000), but using chemicals may cause environmental issues and increase resistance to fungicides (Leroux 2007) whereas the use of some compounds has been restricted and banned in a number of countries. Moreover, quality loss due to gerbera grey mould occurring in post-harvest (in auction or after sale) transport is hard to avoid by chemical treatments, affecting both the buyer (reduction in profits) and the seller (a breeder’s reputation) (Bastiaan-Net et al. 2007). Thus, breeding for *Botrytis* resistant varieties is needed to reduce current and future problems due to this devastating pathogen in gerbera.

A number of studies on *Arabidopsis* indicated that the positive responses regulated by JA/ET (jasmonic acid/ethylene) signalling (Glazebrook 2005; Thomma et al. 1998; Thomma et al. 1999) and production of camalexin (Kliebenstein et al. 2005; van Baarlen et al. 2007; Williamson et al. 2007) enhance a plant’s resistance to *B*. *cinerea*. Similarly, in *Brassica rapa*, glucosinolate defensive metabolite accumulation coincided with *B. cinerea* quantitative trait loci (QTLs) (Zhang et al. 2016). Catechol from onion scales inhibited *B. cinerea* growth in vitro (Clark and Lorbeer 1975)*.* However, none of these metabolites in different species can confer full resistance. Plant resistance to *Botrytis* is considered conditioned by multiple genes with partial effects and likely requires the contribution of multiple loci to reduce disease severity (Mengiste et al. 2003) and to obtain acceptable levels of resistance under standard conditions. This kind of complex resistance is polygenic and can be referred to as quantitative disease resistance (St. Clair 2010). DNA markers tightly linked to quantitative resistance loci can be used for marker-assisted selection (MAS) and desirable QTLs can be then subsequently introgressed into commercial cultivars.

Up to now, QTL analysis for *Botrytis* resistance has been primarily assayed in *Arabidopsis* and tomato. Denby et al. (2004) identified 12 small- to medium-effect QTL governing *Botrytis* susceptibility as to lesion size in *Arabidopsis* using 104 individuals from a Ler × Col-0 recombinant inbred population and several interesting candidate genes were found co-located in the QTL regions of the genome. Rowe and Kliebenstein (2008) found that several separate QTLs influenced lesion size and camalexin accumulation on *Arabidopsis* leaves using a larger RILs population with 411 individuals. They suggested that the plant defence against *B*. *cinerea* is mainly quantitative and genetically complex. Finkers et al. (2007b) calculated disease incidence and lesion growth rate in tomato populations and detected three QTLs that explained 12, 15 and 7% of the total phenotypic variation. They also analysed two QTLs in BC2S1 progeny and found additive effects for progeny with homozygous resistance QTL alleles present.

No gerbera genetic maps are published to date. In this study, we developed two F1 populations segregating for *Botrytis* resistance in order to obtain the first genetic maps for this highly heterozygous ornamental crop. Through next generation sequencing of the transcriptomes of the parental genotypes (Fu et al. 2016), SNP markers have been developed. These SNP markers have been used for linkage map construction and QTL mapping of *Botrytis* resistance in gerbera.

## Materials and methods

### Mapping populations

Two gerbera segregating F1 populations from heterozygous parents were used in this study. The two mapping populations were derived from four parental genotypes with different resistance levels against *B*. *cinerea* infection and the selected (unrelated) two populations showed the largest variation among 20 F1 populations (four half sibs of crosses with a line with known *Botrytis* infection problems) which were tested for *Botrytis* susceptibility on 50 individuals. Population Schreurs (hereafter referred to as population S), containing 276 offspring, was obtained from a cross between the gerbera genotypes, SP1 and SP2. Population Florist (hereafter referred to as population F) was produced by a cross between FP1 and FP2. Population F consisted of 270 progeny. All individuals from both populations were used for linkage mapping, disease tests and QTL analysis.

### Phenotypic measurements

The head-like inflorescence of gerbera is composed of different flower types, the marginal ray florets, the central disc florets and the intermediate trans florets. *Botrytis*-infected lesion symptoms vary in these gerbera florets: spotting on ray florets and rotting on disc florets. To assess *Botrytis* resistance levels on different gerbera inflorescences of all F1 progenies and four parents in the two populations, phenotypic data were collected using three tests based on a visual inspection of *Botrytis* infestation: on whole inflorescence (further referred to as *whole inflorescence* or *WI* test), on the bottom of disc florets in the capitulum (further referred to as *bottom* test) and on ray florets (further referred to as *ray floret* or *RF* test), respectively.

*B. cinerea* (strain B05.10 obtained from Dr. J. van Kan, Laboratory of Phytopathology, Wageningen University) was grown for 1 week on potato dextrose agar (PDA) medium after which conidia were transferred onto fresh PDA medium and grown until sporulation (about 1 week). A spore suspension of 1 × 10^7^ conidial spores per millilitre in sterile distilled water was prepared as stock suspension. For the *Botrytis* disease test on whole inflorescence and bottom, the spore suspension was diluted to a concentration of 1 × 10^5^/ml with water and sprayed on the inflorescence with a fine plant sprayer. After inoculation, inflorescences were incubated for 5 days in a climate cell at 20 °C and a R.H. of 90%. Because ripe flowers (anthesis of first whorl of disc floret) are not available from single plants in abundance, testing was done over a period of 10 consecutive weeks (8–10 inflorescences tested on average). Inflorescence testing was done simultaneously for whole inflorescence and bottom tests on the same inflorescence. First, whole inflorescences were visually evaluated to score, after which, the bottom of the capitulum was cut (horizontal cross section) to check (score) fungal growth inside the capitulum for the bottom test. The response to *Botrytis* infection on whole inflorescence and bottom was scored ranging from 0 (no symptom) to 5 (completely rotten).

For the ray floret test, inoculation was performed by pipetting 2 μl of spore suspension that was diluted to a concentration of 3 × 10^5^/ml in potato dextrose (to guarantee 100% spore germination), on the upper surface of a single marginal ray floret. Twenty ray florets were incubated for 48 h at nearly 100% relative humidity. After 48 h, the disease score was assessed as follows: 0 no visible symptoms; 1 infection limited in inoculation droplet size; 2 lesion extended twice to four times the droplet size; 3 large lesion area but still smaller than half of the ray floret; 4 lesion area larger than half of the ray floret; and 5 complete necrosis.

### SNP selection and genotyping

EST database establishment and SNP detection have been performed as described in Fu et al. (2016). SNPs were identified as specific SNPs (only polymorphic in one set of crossing parents, i.e. a single population) and common SNPs (polymorphic in both populations). The origin of SNP markers is indicated in the name. For example, marker WGC10601_843_S1F2 means that this marker is developed from contig10601 of the gerbera EST data set (Fu et al. 2016). The number after the first underscore is the SNP position in the contig. At the end of each marker’s name, the source of polymorphism is indicated by population (and parent). If it is a specific SNP, it will be followed only with either S or F (S after the second underscore means polymorphic in both S population parents and S1 means only polymorphic in SP1, etc.). Common SNPs are indicated with both an S and F in the name. In this case, S1F2 means this marker is a common marker which is polymorphic in parents S1 and F2 and can be found under an identical name in both maps.

Genotyping of selected reliable SNP markers in parents and all individuals of the two populations was performed by KBioscience (current name LGC genomics) using KASP technology. The genotyping data were visualised in SNPviewer (LGC genomics) to check the segregation type in each population, and SNP markers with segregation type 1:1 and 1:2:1 were included for genetic mapping. SNP makers segregating in a non-Mendelian inheritance pattern were analysed by hypothesising one or more null allele present. After checking the goodness of fit to possible segregation types, these markers were rescored and included.

### Genetic linkage map construction

The genotyping data of two gerbera populations were coded following the population type CP (cross-pollinating) in JoinMap® 4.1 (van Ooijen 2006). After created maternal and paternal population nodes, grouping of markers was based upon the test for independence LOD score with a threshold of 4. Genetic map construction used regression mapping and the Kosambi mapping function. Integrated linkage maps of the parental maps were constructed based on the bridge markers (<hkxhk> type marker). Consensus linkage groups of the two populations were constructed with identical common markers segregating in both populations and numbering was named consistent between linkage maps.

### QTL analysis

The means of disease score for each individual on whole inflorescence, bottom and ray floret tests in the two populations were used independently as phenotypic data for QTL analysis. QTL analysis for *Botrytis* resistance was performed in separate parental linkage maps using MapQTL® 6 (van Ooijen 2009). First, interval mapping was used to find QTL regions associated to each of the traits tested. Based on the result of interval mapping, MQM (multiple QTL models) mapping was performed with the maximum likelihood mixture model using the closest markers as co-factors. Significance LOD thresholds were determined by 1000 permutations corresponding to a genome-wide confidence level of *P* < 0.05.

## Results

### SNP selection and genotyping results

Gerbera cDNA reads were clustered and assembled into 36,770 EST contigs within which a large number of specific and common SNPs were detected in the parents from the two populations (Fu et al. 2016). For genotyping in the population S, a set of 677 polymorphic SNPs markers, including 477 SNPs common to both populations and 200 specific SNPs, was selected. Similarly, there were 675 SNPs markers selected for population F, including 477 common markers and 198 specific SNPs.

A summary of segregation type for all SNP markers in both populations is shown in Table 1. Of all the selected SNP markers, 68% were successfully genotyped by KASP in population S and 72% were successful in population F (Fig. S1a–c illustrate the three visualised segregation results in SNPviewer). A number of markers showing a single group call are considered as non-polymorphic (Fig. S1d); these include 166 SNPs in population S and 147 in population F. Markers showing scattered segregation without clear grouping were noted as not-fitting segregation (Fig. S1e). The percentages of markers showing a not-fitting pattern were 7% in population S and 6% in population F.

**Table 1:** Overview of the genotyping results of selected SNPs marker

Population	Markers segregating in both parents	Markers segregating in P1 (seed parent) only	Markers segregating in P2 (pollen parent) only	Markers with a null allele	No polymorphism	Segregation not fitting
S	135/20%	159/23%	126/19%	41/6%	166/25%	50/7%
F	107/16%	230/34%	116/17%	34/5%	147/22%	41/6%

In a number of cases in both the S and F populations (Table 1, null allele), parental genotype scores do not seem to fit the found offspring genotypes. These segregating SNP markers could be further analysed assuming the presence of null alleles. For example, in marker WGC19112, the genotype of two parents are A:G (P1) and G:G (P2), respectively, and the expected segregation in progeny should be [A:G]:[G:G] = 1:1, but the visualised genotyping result in SNPviewer (Fig. S1f) showed three genotype cluster plots [A:A]:[A:G]:[G:G] ≈ 1:1:2 (74:69:133). The possible explanation is the presence of a null allele in P2 (G:Ø) and the actual progeny segregation is [A:G]:[A:Ø]:[G:G]:[G:Ø] ≈ 1:1:1:1, because the genotyping technology cannot distinguish the genotype G:G and G:Ø (they are in the same cluster), also the P2 genotype G:Ø is recognised as G:G.

To use the marker information, we rescored these markers, like WGC19112, with the consideration that both parents are heterozygous. However, information content differed between the parents for such a marker. P1 is heterozygous and WGC19112 is used as a fully informative <lmxll> marker (a). Both A:G and A:Ø offspring clusters are rescored as “lm” and G:G (in fact containing [G:G] and [G:Ø]) as “ll”. P2 is also heterozygous and WGC19112 is here regarded as <nnxnp> marker (b). However, only offsprings within the groups A:A and A:G are informative for this parent (group A:A scored as “nn” and group A:G as “np”), the mixed group G:G (containing [G:G] and [G:Ø]) is discarded. To distinguish the two ways of scoring, we added a letter “a” or “b” at the end of the marker. Markers in which null alleles were demonstrated with an a and b at the end were mapped at almost the same position on the integrated maps, but in eight markers, sufficient linkage was only found in linkage groups of the most informative parent and not in the other parent.

### Linkage map construction

Both maternal and paternal maps of the two populations were constructed, as well as integrated maps per population and a consensus map of the two populations. There were 30, 29, 27 and 28 linkage groups constructed in SP1, SP2, FP1 and FP2, respectively (Table S1). Total marker number ranged from 259 in parent FP2 to 350 in parent FP1. The observed parental map lengths varied from 1103 to 1498 cM and the average marker distance varied from 3.50 to 4.41 cM per parental map (Table S1).

Parental linkage maps could be aligned via the presence of bridge markers (<hkxhk> type markers) that are segregating from both parents (Table S2). Based on the position of the bridge markers, marker order on parental maps showed good consistency, but the distance between the markers on parental linkage maps varied as can be expected. For instance, on maternal linkage group FP1_08, the distance between the markers WGC9125 and WGC18021 is 7.6 cM, while the distance on the paternal linkage group (FP2_08) is 12.9 cM (Fig. S2a). By using these bridge markers, the two parental linkage maps could be combined into one integrated linkage map with the same linkage group number code (see Fig. S2a).

Similarly, with the help of common markers, identical parental linkage maps of both crosses could be also be identified and aligned. For instance, there are around 35 markers in linkage group SP1_01, of which some markers are also found in two linkage groups in the parents of the F population (i.e. common markers) indicating that these linkage groups are homologues of SP1_01. So, these fragments are named as FP1_01.1 and FP1_01.2 and as FP2_01.1 and FP2_01.2. The same situation happens on SP1_03, SP2_03, SP1_12, SP2_12, etc. (Table S1). Few maternal and paternal linkage groups (e.g. SP1_21, SP2_23 and FP1_17, FP1_24, FP2_16, FP2_20) could not be aligned to a linkage group of another parent because just a single bridge marker was present or there was a lack of informative common markers (Tables S1 and S2).

**Fig. 1:**
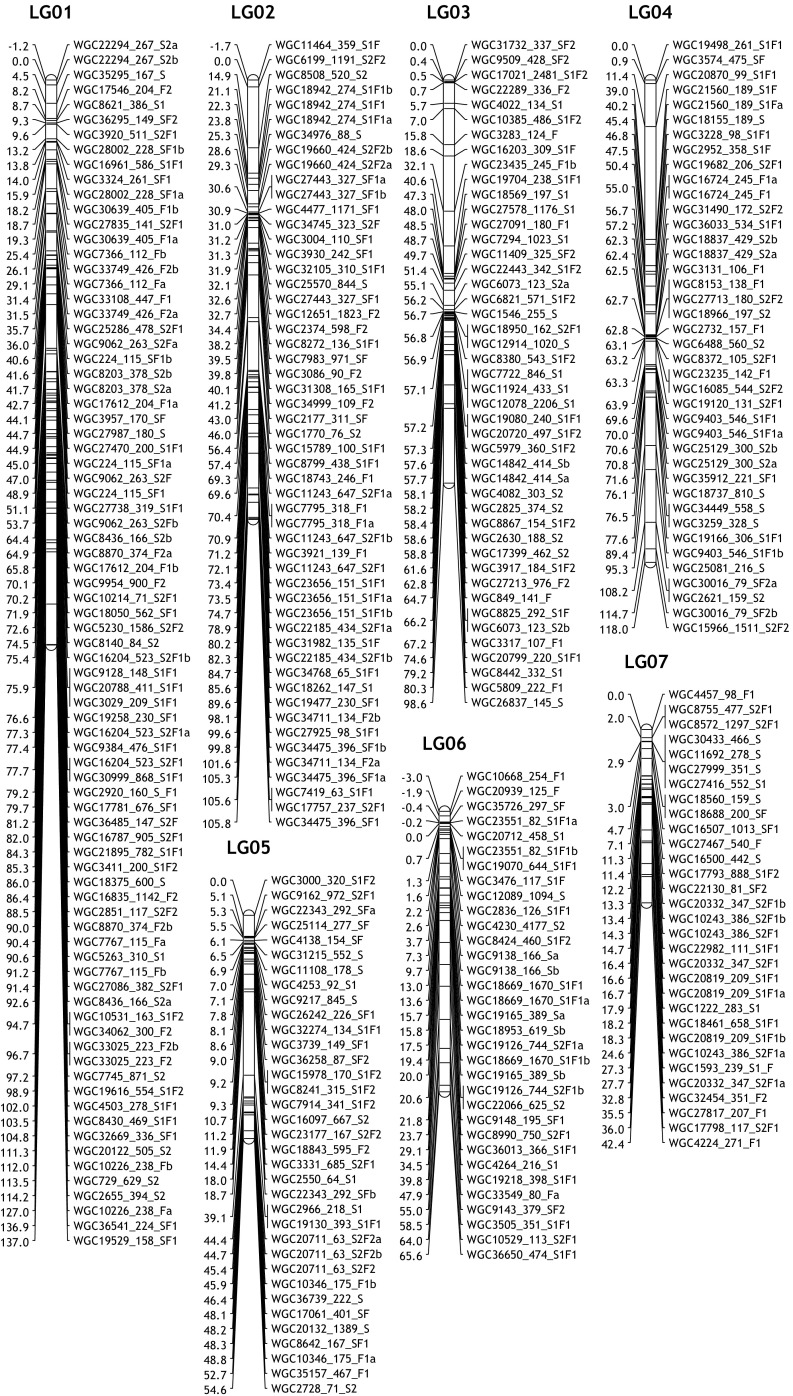

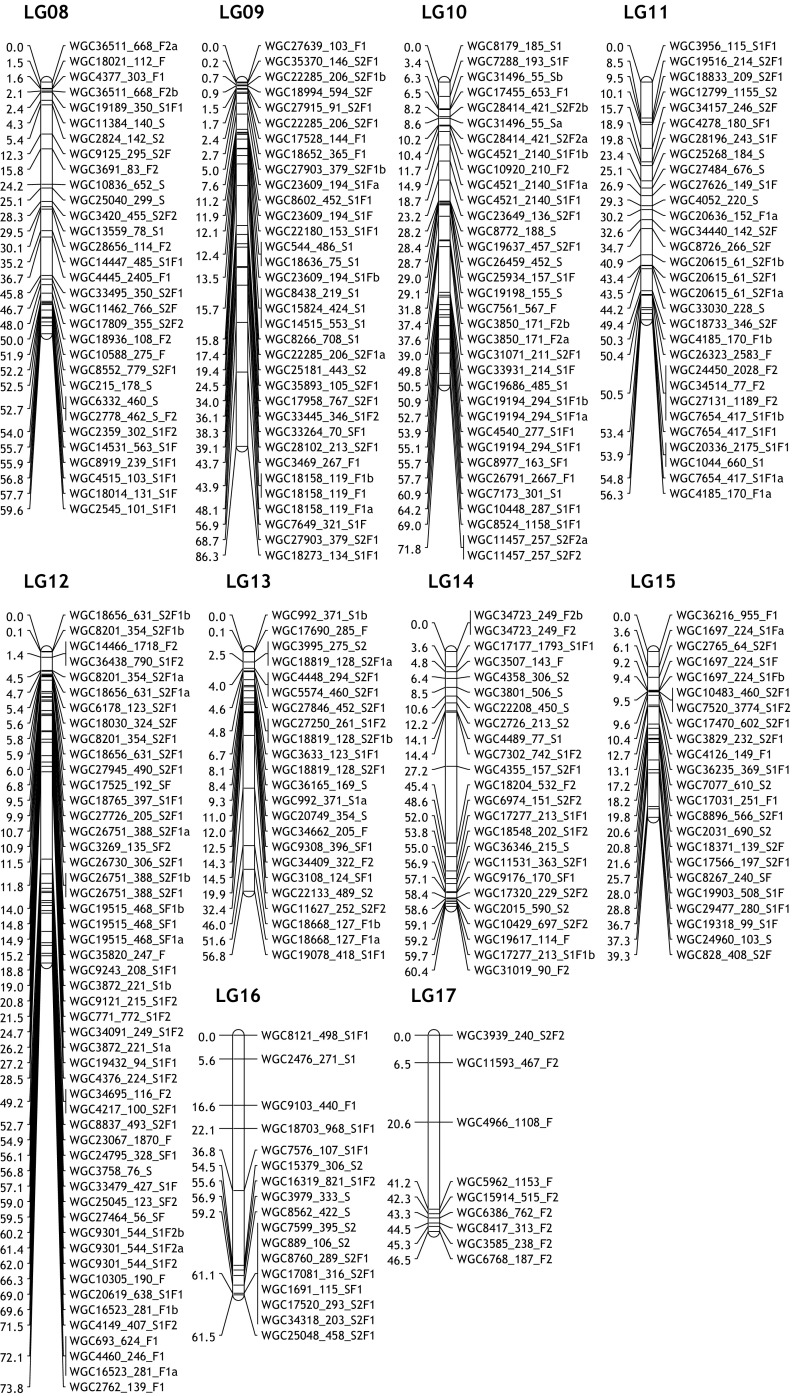
Consensus linkage map of gerbera

These parental linkage maps, with a total of 285 common markers present, can be integrated into a consensus map (see Fig. S2b). In total, 24 consensus linkage groups were merged (Fig. 1, Table S3). As is described in Table S3, the consensus linkage map of 687 SNPs covered 1601 cM. The marker density on the consensus map varied from 1.32 cM on linkage group 09 (LG09) to 5.16 cM on LG17, with an average density of 2.57 cM. There were 14 gaps larger than 15 cM observed in the consensus linkage map.

### Phenotypic traits evaluation for *Botrytis* resistance

**Fig. 2:**
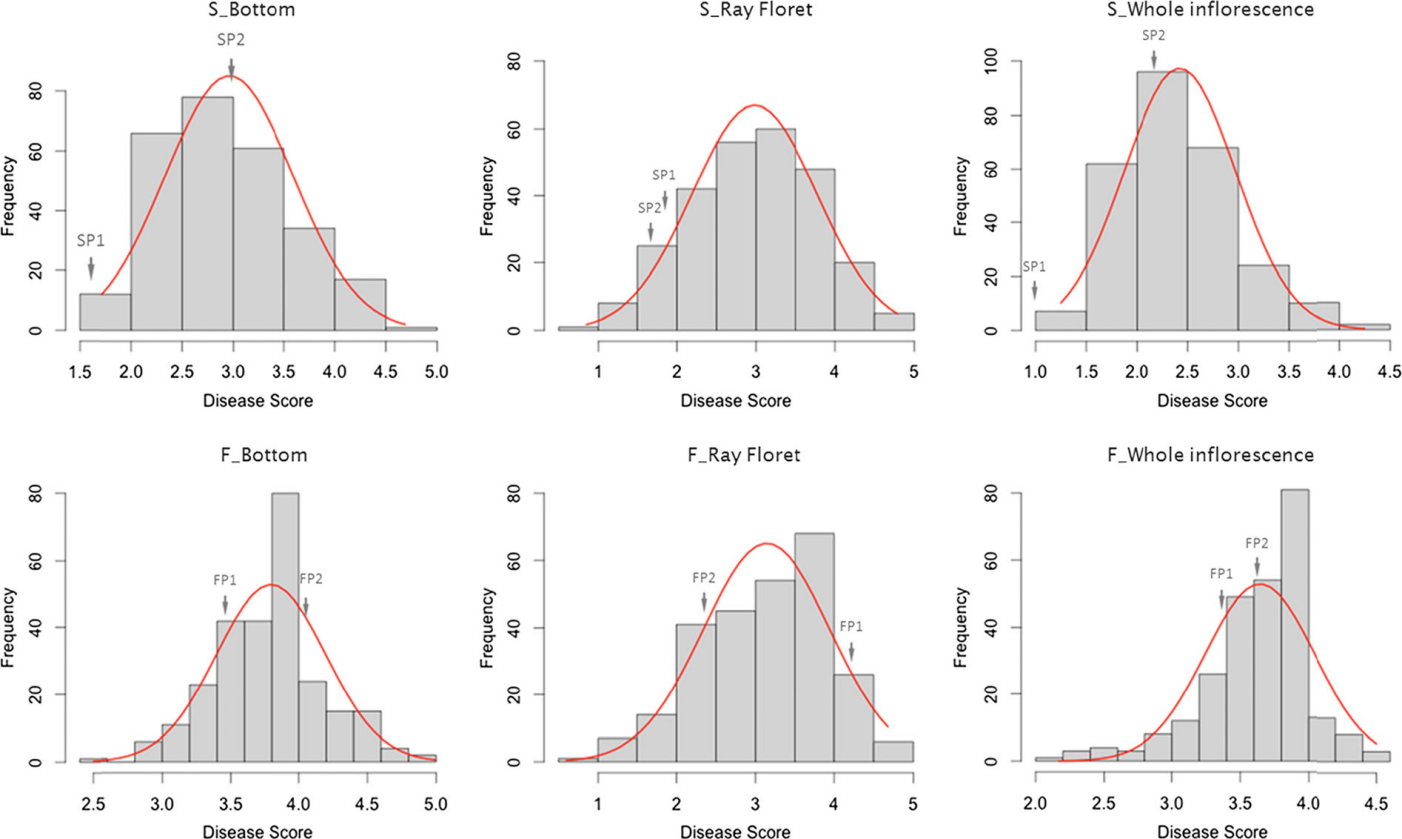
Disease index distribution of *S* and *F* populations in *bottom*, *ray floret* and *whole inflorescence*, respectively. Normal distribution curves are shown above the histograms in *red*. *Arrows* indicate the disease score of parents

Phenotypic data of resistance to *B. cinerea* were assessed in three tests (whole inflorescence, bottom and ray floret). Histograms of disease testing, resulting from these three traits in the mapping populations S and F and indicating transgressive segregations are shown in Fig. 2. The means of the phenotyping data in population S for whole inflorescence, bottom and ray floret were 2.42 ± 0.55, 2.96 ± 0.63 and 2.98 ± 0.79. Means in population F were 3.64 ± 0.40, 3.80 ± 0.40 and 3.14 ± 0.80, respectively. Based on the skewness and kurtosis scale of the distribution curves, all three tests in the two populations were considered as approximately normally distributed and no transformation of data was performed for QTL analysis.

Disease index of the three disease tests in both populations was analysed by Pearson correlation (Table S4). The coefficients of whole inflorescence and bottom tests in both populations showed moderately high correlations (*R* = 0.83 in population S, *R* = 0.67 in population F), but no significant correlation was found to the ray floret tests.

### QTL analysis

QTL analysis was first performed on the four parental linkage maps individually. The genome-wide (GW) LOD significance thresholds (*P* < 0.05) for whole inflorescence, bottom and ray floret were obtained using a permutation test (Table 2). Markers, with LOD scores above the GW threshold in every QTL after interval mapping (IM), were chosen as co-factors for multiple QTL models mapping (MQM mapping). Significant QTLs detected from the four parents are shown in Table 2 and Fig. S3a–c.

**Table 2:** QTLs found for whole flower, bottom and petal test in the parental genotypes of both populations

QTL	Parents	Flanking markers	LG	MQM	
				LOD (GW)	% expl
RBQB1	SP1	WGC11243_647_S2F1a	2	4.4 (4.0)	6.3
RBQB2	SP1	WGC2476_271_S1	16	4.6 (4.0)	6.6
RBQB3	SP2	WGC18733_346_S2F	11	4.6 (4.1)	7.6
RBQB4	FP1	WGC16204_523_S2F1	1	6.8 (4.0)	10.3
RBQB5	FP1	WGC28102_213_S2F1	9	4.5 (4.0)	7.5
RBQB6	FP2	WGC18158_119_F1b	9	4.8 (3.9)	8
RBQRF1	SP2	WGC17798_117_S2F1	7	5.3 (4.0)	8.9
RBQRF2	FP1	WGC22343_292_SFa	5	6.5 (4.1)	8.6
RBQRF3	FP1	WGC35370_146_S2F1	9	4.8 (4.1)	6.2
RBQRF4	FP1	WGC828_408_S2F	15	6.1 (4.1)	8
RBQRF5	FP1	WGC35264_283_S2F1	18	5.9 (4.1)	7.6
RBQRF6	FP2	WGC7520_3774_S1F2	15	4.9 (4.0)	7
RBQRF7	FP2	WGC6074_441_S2F	18	4.0 (4.0)	5.7
RBQRF8	FP2	WGC9226_226_F2	21	4.75 (4.0)	8.0
RBQWI1	SP1	WGC1044_660_S1, WGC33030_228_S	11	4.8 (4.0)	7.3
RBQWI2	SP1	WGC407_4995_S1F1	23	5.3 (4.0)	8.2
RBQWI3	SP2	WGC18733_346_S2F	11	5.2 (4.1)	8.6
RBQWI4	FP1	WGC1084_721_F	23	6.8 (4.1)	11.1
RBQWI5	FP2	WGC5962_1153_F	17	5.6 (4.0)	8.3
RBQWI6	FP2	WGC22447_285_Fb	23	7.6 (4.0)	11.4

Name of QTLs are RBQ (as Resistance Botrytis QTL) followed by the initials of disease tests used. Null alleles are marked with a letter ‘a’ or ‘b’ in the end

*B* Bottom, *PF* ray floret, *WI* whole inflorescence test, *LG* indicates linkage group and the LG number in the two populations, *GW* indicates genome-wide significant threshold level *P* < 0.05; % expl. is the percentage of total variance explained by the QTL

In the S population, seven significant QTLs for *Botrytis* resistance were detected by MQM mapping and 13 QTLs in the F population. The difference in numbers of QTLs found between the two populations is defined by the number of QTLs associated with *Botrytis* resistance in ray floret. There is only one ray floret QTL found in the S population but seven in the F population (Table 2). Phenotypic variance explained by single QTLs ranged between 5.7 and 11.4%, with three QTLs (RBQB4, RBQWI4 and RBQWI6) higher than 10%. Three QTLs, RBQWI2, RBQWI4 and RBQWI6 from SP1, FP1 and FP2, respectively, were found on LG23 at similar positions in the consensus map (see Fig. S4) indicating this may be a single QTL. Interestingly, a QTL for whole inflorescence and bottom (RBQB3 and RBQWI3) shared an identical position with marker WGC18733_346_S2F on LG11 of population S.

Several QTLs were detected on both parental linkage groups separately and showed overlapping positions on the integrated linkage group, like RBQWI1 from SP1 and RBQWI3 from SP2 on LG11 and RBQB5 from FP1 and RBQB6 from FP2 on LG9. In these cases, alleles from both parents contributed to the resistance in the progeny. We identified the favourable and unfavourable alleles from the parents of these QTLs. Progeny can then be divided into four groups: progeny with the presence of two favourable alleles (+/+), with one favourable allele from one of the parents (+/− or −/+) and no favourable allele present (−/−). The mean disease score of each progeny group for each QTL is shown in Table 3. The mean disease scores of individuals with two favourable alleles (+/+) were all significantly lower than those for individuals with no favourable allele present (−/−) and also show advantage over individuals with one favourable allele only.

**Table 3:** Difference between the mean score of individuals with the presence of two, one or no favourable allele from the parents

	Bottom	Ray floret		Whole inflorescence	
QTLs	RBQB5 + RBQB6	RBQRF4 + RBQRF6	RBQRF5 + RBQRF7	RBQWI1 + RBQWI3	RBQWI4 + RBQWI6
Flanking markers	WGC28102_213_S2F1	WGC828_408_S2F	WGC35264_283_S2F1	WGC33030_228_S	WGC1084_721_F
	WGC18158_119_F1b	WGC7520_3774_S1F2	WGC6074_441_S2F	WGC18733_346_S2F	WGC22447_285_Fb
Genotype^1^	Mean ± S.E.	Mean ± S.E.	Mean ± S.E.	Mean ± S.E.	Mean ± S.E.
+/+	3.621 ± 0.044^a2^	2.840 ± 0.099^a^	2.732 ± 0.126^a^	2.244 ± 0.060^a^	3.436 ± 0.057^a^
+/−	3.847 ± 0.041^bc^	3.328 ± 0.090^b^	3.152 ± 0.071^b^	2.440 ± 0.067^b^	3.631 ± 0.043^b^
−/+	3.764 ± 0.058^b^	3.299 ± 0.096^b^	3.152 ± 0.071^b^	2.316 ± 0.057^ab^	3.739 ± 0.050^bc^
−/−	3.938 ± 0.052^c^	3.414 ± 0.121^b^	3.430 ± 0.115^b^	2.635 ± 0.074^c^	3.776 ± 0.041^c^

^1^+/+ represents individuals with the presence of two favourable alleles from both parents; +/− represents individuals carrying one favourable from P1 and one unfavourable allele from P2; −/+ represents individuals carrying one unfavourable allele from P1 and favourable from P2; −/− represents individuals with the presence of the two unfavourable alleles from both parents

^2^mean of each groups with letter a, b and c shows significant difference (*P* < 0.05)

## Discussion

### Genetic linkage mapping and integration

In this study, we constructed the first gerbera genetic linkage maps from two F1 populations using newly generated SNP markers. Genetic linkage map construction for cultivated ornamental crops often use F1 populations (Debener and Mattiesch 1999; Han et al. 2002; Rajapakse et al. 2001; Shahin et al. 2011; Zhang et al. 2010) because many ornamental plants, including gerbera, are outcrossing species with complex genetic backgrounds and high heterozygosity that cannot be easily selfed due to serious inbreeding depression effects.

Four parental genetic linkage maps were constructed by using SNP markers from EST data (Fu et al. 2016). Most selected SNP markers showed a Mendelian segregation in the populations. For some loci, the allele segregation and allele ratios indicated the presence of null alleles. Three flanking markers of a QTL contained null alleles. These markers with null alleles probably come from mutations in the marker region which in the RNAseq data analysis of Fu et al. (2016) may have led to assembly of these sequences in alternative contigs and thus stayed unnoticed during SNP identification. We scored markers with null alleles for each parent separately in order to use the marker data as much as possible and found these two-way scored markers are mapped on almost the same positions in the integrated map and the consensus map. The fact that the four alleles of these markers are all different reflects the complex genetic background of gerbera.

Based on the location of the bridge (<hkxhk> type) markers on the maternal and paternal linkage maps, we found the markers’ order on parental maps shows a good consistency, but the distance between the markers on parental linkage maps varies. This is caused by independent meiotic events occurring in the two heterozygous parents and the different frequency of recombination determines the location of markers in each parent (Gebhardt 2007). This also explains the difference in linkage group length between the parents of a cross. Markers common to both populations could be used to merge maps between the two populations and to arrive at a consensus map which was helpful for comparisons between the two populations in QTL mapping. From the integrated and consensus maps, we notice that some parental chromosomes appeared as separate (fragmented) linkage groups in one genotype whereas they were in one LG in another genotype (e.g. FP1_01.1 and FP1_01.2 vs SP1_01). Fragmentation also occurs in integrated maps of single populations. Generally, this occurs more often in the F population (9 out of 20 integrated LGs) than in the S population (6 out of 21 integrated LGs). This might be due to a lower number of markers in FP2. Given the offspring numbers in both populations, a theoretical minimum marker distance of 0.4 cM is possible. So, by introducing more markers, map quality may be further improved.

For gerbera, we expected 25 linkage groups (2*n* = 50). However, a total of 24 consensus linkage groups could be established. There are no additional linkage groups left in any of the four parental maps which could be assigned to LG25. This could be related to the size of this particular chromosome and the number of markers used in our study. Introducing higher numbers of markers might result in retrieving LG25. Also, a lack of polymorphism between alleles of this chromosome could cause the inability to find this linkage group.

### Gerbera grey mould phenotyping

Gerbera grey mould occurs mainly on gerbera capitulum in the production and post-harvest process. Different symptoms in infected gerbera cultivars were found, either necrotic spots on ray and trans florets or rot on disc florets. The mechanism underlying plant resistance against *B*. *cinerea* is not well understood, but it is generally accepted that plant resistance to this necrotrophic pathogen is quantitative and polygenic (Poland et al. 2009; Rowe and Kliebenstein 2008; St. Clair 2010). In a structured mapping population, quantified disease indexes after inoculation can be used to analyse plant responses to this pathogen and perform QTL mapping. However, there is no standard bioassay approach for evaluating plant resistance to *B*. *cinerea* available.

Previous studies on *Arabidopsis* and tomato (AbuQamar et al. 2006; Denby et al. 2004; Ferrari et al. 2007; Finkers et al. 2008; Finkers et al. 2007b; Hu et al. 2013; Rowe and Kliebenstein 2008; ten Have et al. 2007; Zhang and Van Kan 2013) are mainly based on infection assays using drop inoculation or spray inoculation with conidia suspension on leaves or stems, then measuring the lesion expansion rate, lesion size or camalexin accumulation. In gerbera, leaf and stem infections are of little importance and mainly flower infections lead to losses. To thoroughly assess the disease severity on gerbera flowers, we developed spray-inoculation tests on whole inflorescences (whole inflorescence and bottom), as well as a droplet-inoculation test for single ray florets. The tests were devised as simple tests in which a large number of flowers and ray florets could be tested in a relatively short period of time to avoid season influences during the testing period.

As a necrotrophic pathogen, *Botrytis* relies primarily on its abilities to kill the host plant cell and subsequently decompose the plant tissue and consume it for its own growth (van Kan 2006). The fungus can use different infection paths in the complex organs that capitulum are and from experiments with cultivar panels, different responses between cultivars were observed and these led to the three different tests used. Among the three tests, there is a high correlation in both populations between the infection data of whole inflorescence and bottom disease symptoms. Apparently, the mechanism of defence within the chosen parents of the two crosses is more similar with regard to these two traits compared to the wider set of cultivars used in the development of the tests. The test on ray florets (petal) is clearly different from the other two tests. Therefore, for the ray floret test versus whole inflorescence and bottom, it can be envisaged that different genes are involved in resistance to *Botrytis*. Similarly, ten Have et al. (2007) also observed that resistance to *Botrytis* on tomato leaves and stems is distinct from each other.

### QTLs mapping and analysis

QTLs detected varied between the two populations and also between the three tests. The reason for the lower number of QTLs found in the S population for ray floret resistance might be the small difference in ray floret disease score between the two parents of this population. The two F population parents, by contrast, showed a large difference in disease score for ray floret. Three QTL regions for whole inflorescence test, which were detected on different parental maps separately, co-localised on linkage group 23 for both populations. Although the QTL region still spans 20 cM on the consensus map between the most significant loci, the flanking bridge markers indicated possible overlapping of parental linkage groups and the existence of favourable alleles.

A relative high correlation between bottom and whole inflorescence was found in both populations, yet there is only one identical locus in both populations showing a significant QTL in both tests. More common QTLs for whole inflorescence and bottom might be expected given the correlation between the two tests. Apparently, not all QTLs underlying the high correlation of the two tests can be detected which could be due to a lack of resolving power to detect minor QTLs for both disease indexes at the same time in a population. Environmental variance between test weeks may influence both tests in a similar way; however, numbers of repetitions per week were too low to be able to study this.

In this study, several QTLs with minor effect for *Botrytis* resistance on gerbera inflorescences were detected. The results showed that, similar to *Botrytis* resistance in other plants, defence against *B*. *cinerea* on gerbera is quantitative and genetically complex, with probably the involvement of different infection mechanisms (Denby et al. 2004; Finkers et al. 2007a; Rowe and Kliebenstein 2008). QTLs found in our study may seem minor-effect QTLs, which are more difficult to use in breeding programs than major-effect QTLs or single resistance genes. However, several QTLs detected from separate parental linkage groups were found in overlapping locations on the integrated map and we assume that these correlative QTLs are probably from a common gene with positive and negative alleles which can be defined as quantitative trait alleles (QTAs, Schäfer-Pregl et al. 1998). With the presence of two positive QTAs, gerbera resistance to *Botrytis* increased significantly.

For two reasons, we think there is potential for introgression of favourable QTL alleles in breeding to increase resistance to *Botrytis* in gerbera: (a) phenotyping *Botrytis* disease is difficult and the large environmental component in testing has a downsizing effect on the total explained variance found in QTLs, i.e. contributions of QTLs to genetic variance explained will be higher and (b) compared to the disease tests (conditions chosen to avoid effects of incidence), the disease pressure in commercial greenhouses will be much lower and environmental conditions are less favourable for *Botrytis* infection (Finkers et al. 2007b). Under such conditions, the effect of the QTLs may be much stronger.

This mapping study provides the first genetic map of gerbera and by using SNP markers derived from EST sequences (Fu et al. 2016), a generally useable framework is provided which can be used for other studies as well and provides the first step in unravelling the complexity of the genetic background of resistance to *Botrytis* in gerbera.

## Electronic supplementary material

Fig. S1 (DOCX 939 kb)

Fig. S2 (DOCX 104 kb)

Fig. S3 (DOCX 474 kb)

Fig. S4 (DOCX 46 kb)

ESM 1 (XLSX 23 kb)
