# Supplementary material for: Genetic mapping and QTL analysis of Botrytis resistance in Gerbera hybrida
Source: Mol Breed. 2017 Jan 23;37(2):13. doi: 10.1007/s11032-016-0617-1 (PMC5285436; doi:10.1007/s11032-016-0617-1)
Supplement: Supplementary file 1 — (DOCX 939 kb) [file 11032_2016_617_MOESM1_ESM.docx]

**Fig. S1.** Examples of different segregation types for SNP markers visualized by SNPviewer. **a**: the parental genotypes are T:A indicating a <hkxhk> marker, and the progeny genotypes are T:T (n=66), T:A (n=140) and A:A (n=69) segregating in the ratio 1:2:1. **b**: the first parent genotype is A:G, and the second is are G:G indicating a <lmxll> marker. The progeny genotypes are A:G (n=135) and G:G (n=141) segregating in the ratio 1:1. **c**: the first parent genotype is C:C, and the second is are T:C indicating a <nnxnp> marker. The progeny genotypes are C:C (n=133) and T:C (n=134) segregating in the ratio 1:1. **d**: not polymorphic marker. Two parents and all progeny are in the same A:A cluster without segregation. **e**: not-fitting segregation. The parental genotypes are G:A, and the progeny should be segregating in 1:2:1 ratio but in fact they are scattered and no clear clusters can be defined. **f**: non Mendelian segregating marker with putative null-allele. Parents seem to be A:G and G:G, whereas progeny show clusters A:A, A:G and G:G with 74, 69 and 133 individuals respectively indicating that the true genotype of parent P2 is likely G: Ø.
