# Supplementary material for: Genetic mapping and QTL analysis of Botrytis resistance in Gerbera hybrida
Source: Mol Breed. 2017 Jan 23;37(2):13. doi: 10.1007/s11032-016-0617-1 (PMC5285436; doi:10.1007/s11032-016-0617-1)
Supplement: Supplementary file 2 — (DOCX 104 kb) [file 11032_2016_617_MOESM2_ESM.docx]

**Fig. S2.** a: Parental and integrated map of LG08 from F population. Identical markers are linked. The anchor markers (<hkxhk> type loci) are highlighted in red. b: Integrated and consensus map (in the middle) of LG08 of the two populations. Bridge (common) markers of S and F population are indicated in red.
