# Supplementary material for: Genetic mapping and QTL analysis of Botrytis resistance in Gerbera hybrida
Source: Mol Breed. 2017 Jan 23;37(2):13. doi: 10.1007/s11032-016-0617-1 (PMC5285436; doi:10.1007/s11032-016-0617-1)
Supplement: Supplementary file 3 — (DOCX 474 kb) [file 11032_2016_617_MOESM3_ESM.docx]

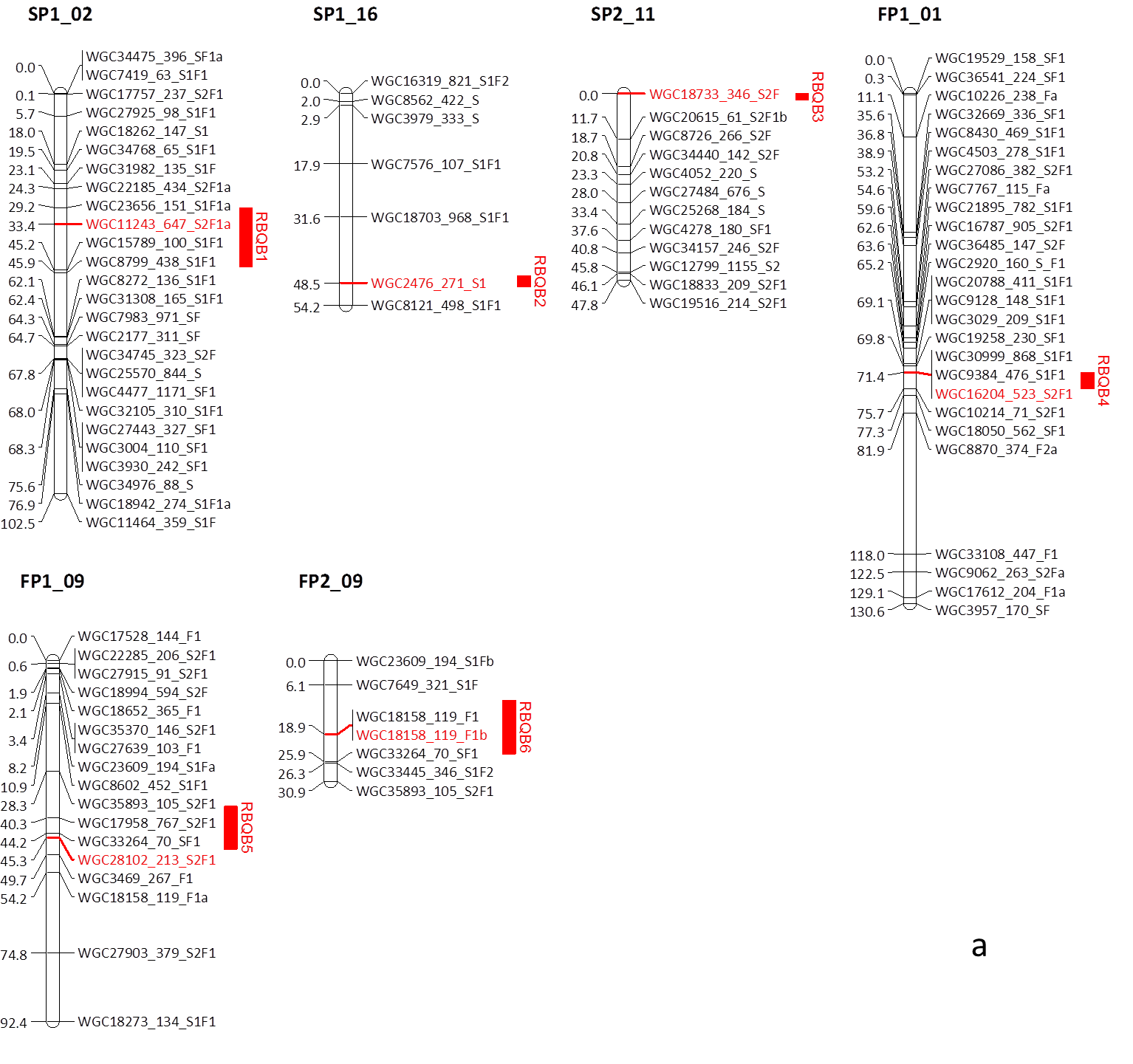


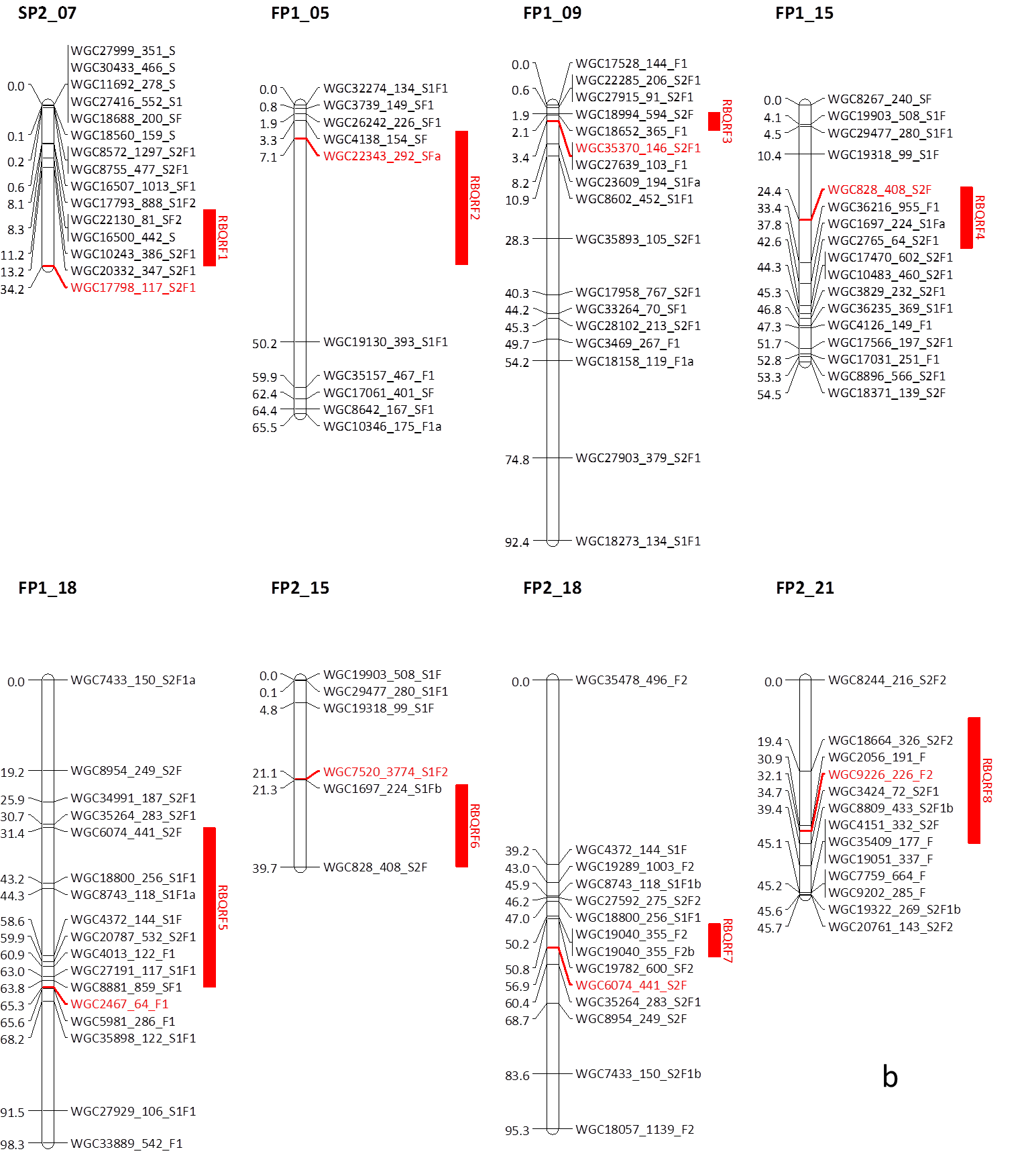


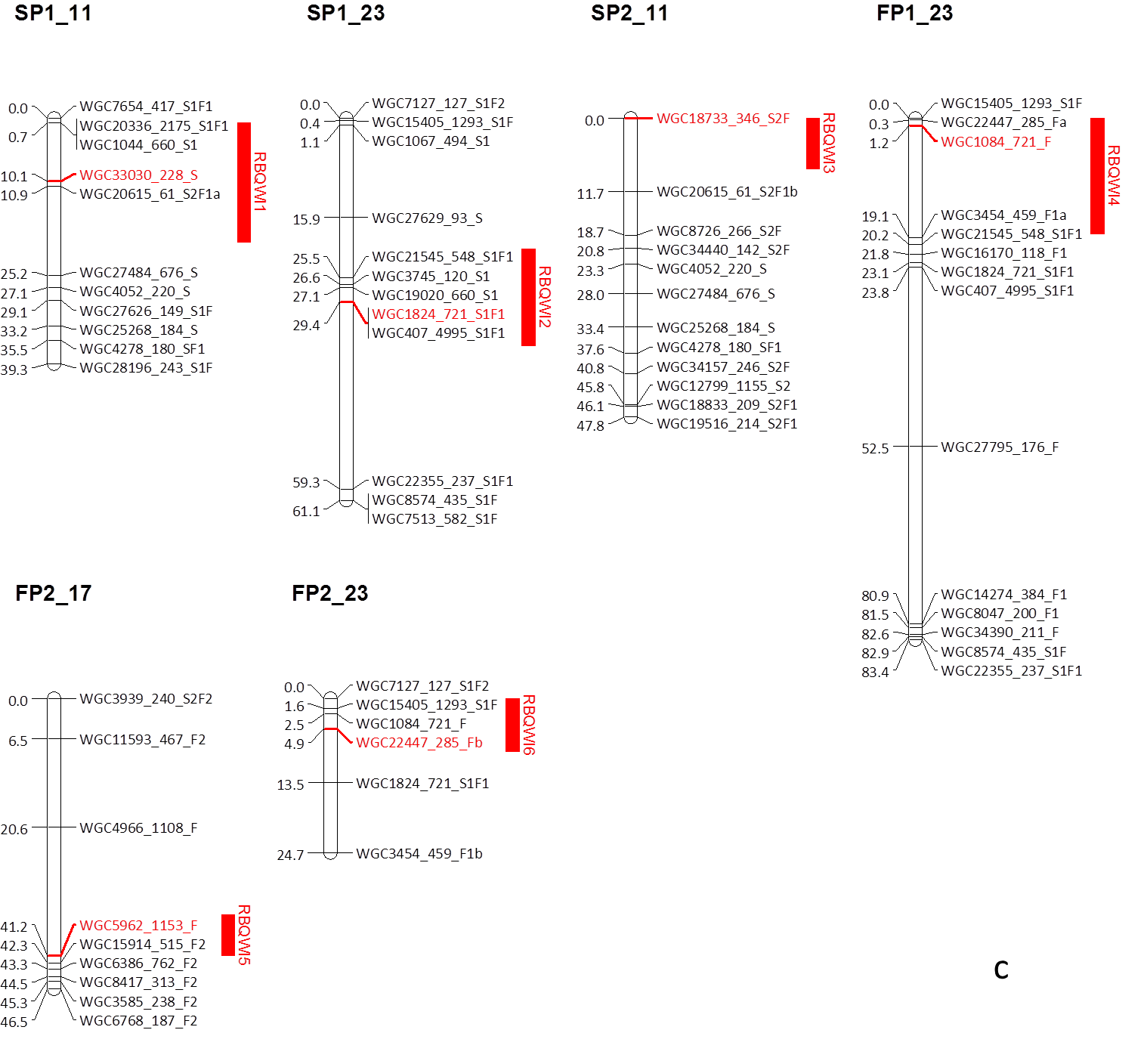


**Fig. S3.** a: QTLs locations in parental linkage maps for *bottom* test. b: QTLs locations in parental linkage maps for *ray florets*  test. c: QTLs locations in parental linkage maps for *whole inflorescence* test. Red bars represent the LOD 95% confidence intervals for QTL peaks and red markers represent the QTL peak location.
