# Supplementary material for: Genetic mapping and QTL analysis of Botrytis resistance in Gerbera hybrida
Source: Mol Breed. 2017 Jan 23;37(2):13. doi: 10.1007/s11032-016-0617-1 (PMC5285436; doi:10.1007/s11032-016-0617-1)
Supplement: Supplementary file 4 — (DOCX 46 kb) [file 11032_2016_617_MOESM4_ESM.docx]

**Fig. S4.** The location of QTLs (RBQWI2, RBQWI4 and RBQWI6) on parental linkage maps. The bars in red colour show the position of the QTL for *whole inflorescence in* three different parental linkage maps and **, ****, ****** indicated significant at P=0.05, 0.005, 0.0005 of Kruskal–Wallis test. The highest marker QTL is in red marker and common markers are underlined.
